# Overloaded axial stress activates the Wnt/β-Catenin pathway in nucleus pulposus cells of adult degenerative scoliosis combined with intervertebral disc degeneration

**DOI:** 10.1007/s11033-023-08390-9

**Published:** 2023-04-08

**Authors:** Zhijun Cai, Qibiao Luo, Xi Yang, Luqiao Pu, Haiyang Zong, Rongmao Shi, Pengju He, Yongqing Xu, Yang Li, Jianping Zhang

**Affiliations:** Department of Orthopedics, The People’s Liberation Army Joint Logistic Support Force 920th Hospital, No. 212 Daguan Rd, Kunming, Yunnan 650032 China

**Keywords:** Degenerative scoliosis, Intervertebral disc degeneration, Nucleus pulposus cells, Axial stress, Wnt/β-Catenin pathway

## Abstract

**Background:**

Intervertebral disc degeneration (IVDD) is the initiating factor of adult degenerative scoliosis (ADS), and ADS further accelerates IVDD, creating a vicious cycle. Nevertheless, the role of the Wnt/β-Catenin pathway in ADS combined with IVDD (ADS-IVDD) remains a mystery. Accordingly, this study was proposed to investigate the effect of axial stress on the Wnt/β-Catenin pathway in nucleus pulposus cells (NPCs) isolated from DS-IVDD patients.

**Methods:**

Normal NPCs (N-NPC) were purchased and the NPCs of young (25–30 years; Y-NPC) and old (65–70 years; O-NPC) from ADS-IVDD patients were primary cultured. After treatment of NPC with overloaded axial pressure, CCK-8 and Annexin V-FITC kits were applied for detecting proliferation and apoptosis of N-NPC, Y-NPC and O-NPC, and western blotting was performed to assess the expression of Wnt 3a, β-Catenin, NPC markers and apoptosis markers (Bax, Bcl2 and Caspase 3).

**Results:**

N-NPC, Y-NPC and O-NPC were mainly oval, polygonal and spindle-shaped with pseudopods, and the cell morphology tended to be flattened with age. N-NPC, Y-NPC and O-NPC were capable of synthesizing proteoglycans and expressing the NPC markers (Collagen II and Aggrecan). Notably, the expression of Wnt 3a, β-Catenin, Collagen II and Aggrecan was reduced in N-NPC, Y-NPC and O-NPC in that order. After overload axial stress treatment, cell viability of N-NPC and Y-NPC was significantly reduced, and the percentage of apoptosis and expression of Wnt 3a and β-Catenin were significantly increased.

**Conclusions:**

Overloaded axial pressure activates the Wnt/β-Catenin pathway to suppress proliferation and facilitate apoptosis of NPC in ADS-IVDD patients.

**Supplementary Information:**

The online version contains supplementary material available at 10.1007/s11033-023-08390-9.

## Introduction

Adult degenerative scoliosis (ADS) is a spinal deformity with a Cobb angle > 10° in the coronal plane of the spine that develops in adulthood [1, 2]. According to statistics [3] the morbidity rate of ADS is about 37.6%, and the incidence rate is higher in women than in men, with an increasing trend with age. The underlying pathogenesis of ADS is still unclear. Most scholars believe that intervertebral disc degeneration (IVDD) plays an important initiating and central role in the pathogenesis of ADS [4–6]. The intervertebral disc (IVD) consists of a soft central nucleus pulposus, a tough annulus fibrosus, and a hyaline cartilage endplate, which acts as a shock absorber in the spine and allows for spinal motion. Studies have demonstrated that with aging, changes such as an increase in protease activity and a decrease in proteoglycan synthesis led to a reduction in the height and mechanical properties of the IVD, which contributed to the onset and development of ADS [7]. In turn, ADS can further aggravate IVDD [8], which forms a vicious circle. Therefore, there is an urgent need to investigate the mechanisms of occurrence and development of IVDD in ADS.

IVDD is the result of a combined effect of several factors, such as biomechanics, genetics, nutritional deprivation, and inflammation, among which excessive stress is considered to be the primary causative factor of IVDD [9, 10]. The main biological forces present in IVD including axial stress, shear stress and hydrostatic pressure, etc., where axial stress is assumed to be the main factor in the initiation and deterioration of IVDD [11, 12]. However, some studies have shown that IVDD is weakly correlated with physical activity [13, 14]. It is currently believed that the interaction of cellular, extracellular matrix (ECM) and biological forces maintains the homeostasis of IVD. Abnormal biomechanics reduce or terminate proteoglycan and collagen II production in nucleus pulposus cells (NPC), which would decrease hydrostatic pressure leading to progressive degeneration of IVD [12, 15]. Classical Wnt/β-catenin pathway is an important component of the intracellular signaling pathway, and is involved in the regulation of multiple cellular functions such as proliferation, differentiation and regeneration [16, 17]. Classical Wnt/β-catenin pathway is not only intimately involved in the formation and development of IVD, but also in the regulation of IVDD [18]. It was revealed that activation of Wnt/β-catenin pathway promotes TNF-α signaling to mediate the expression of matrix Metalloproteinases (MMPs), leading to a decrease in collagen II and proteoglycan synthesis, which exacerbates the degenerative process [19]. Moreover, cytokines and inflammatory factors that play an important role in IDD interact with the Wnt/β-catenin pathway to synergistically promote IVDD [20, 21]. Similarly, PKC signaling in the non-classical Wnt/β-catenin pathway has been shown to be involved in autophagy, ECM remodeling and oxidative stress of NPC in IVDD [22–24]. Nevertheless, it remains to be investigated whether axial stress is involved in regulating proteoglycan and collagen II production through modulating the Wnt/β-catenin pathway in ADS combined with IVDD (ADS-IVDD).

Based on this, we proposed to isolate NPCs from ADS-IVDD patients of different ages and mediate NPCs by overloaded axial stress for exploring the changes in Wnt/β-catenin pathway activity in different NPCs, and the effect of overloaded axial pressure on Wnt/β-catenin pathway activity. It aims to elucidate the molecular mechanism of axial stress-mediated IVDD development in ADS.

## Materials and methods

### Clinical samples

The patients were diagnosed with ADS and IVDD according to spinal magnetic resonance and Pfirrmann grading criteria. Inclusion criteria: ① patients with confirmed diagnosis of ADS-IVDD with typical clinical features of scoliosis and disc degeneration; ② patients requiring surgical resection of IVD; ③ patients with Pfirrmann grading criteria of grade 3 or higher. Exclusion criteria: ① patients with spinal tumors and infections; ② patients with combined systemic chronic wasting diseases; ③ patients with severe malnutrition or autoimmune diseases. ④ Women during lactation and pregnancy. Ten cases of NP tissues were collected from young and old patients with ADS-IVDD at the People’s Liberation Army Joint Logistic Support Force 920th Hospital. The tissues involved in this study were consented by the patients or their families, who also signed the informed consent form. The study was approved by the ethics committee of the People’s Liberation Army Joint Logistic Support Force 920th Hospital. The clinical characteristics of the patients were shown in Table 1.

**Table 1:** Clinical information of patients

Number	Gender	Age	Pathology	Pfirrmann grade	VAS grade
1	Male	56	DLS, LSS, LDH	IV	6
2	Male	60	DLS, LDH	IV	6
3	Female	58	DLS	IV	5
4	Male	69	DLS, LSS, LDH	V	6
5	Male	68	LSS, LDH	IV	6
6	Male	30	DLS, LDH	IV	5
7	Female	24	LSS, LDH	III	5
8	Male	28	LSS	IV	6
9	Male	26	LSS, LDH	III	5
10	Male	25	DLS	IV	5

The above patients all suffered from adult degenerative scoliosis and intervertebral disc degeneration. DLS stands for degenerative lumbar spondylolisthesis; LSS indicates lumbar spinal stenosis; LDH represents lumbar disc herniation.

### Primary culture and identification of NPC

Nucleus pulposus tissues from young and old ADS-IVDD patients were isolated for young (Y-NPC) and old (O-NPC) NPCs, respectively. The fibrous and bone-like tissue surrounding the nucleus pulposus was removed with ophthalmic forceps (Cofoe, Germany). After three pre-chilled PBS (ZSGB-Bio, China) rinses, the nucleus pulposus tissue was prepared into 1 mm^3^ tissue blocks with sterile ophthalmic scissors (Cofoe, Germany) and placed in DMEM/F12 culture medium (Thermo Fisher Scientific, USA). The tissue blocks were mixed with 0.25% trypsin (Solarbio, China) in a ratio of 1:6 and digested in a water bath for 20–30 min. After digestion, the mixture was centrifuged at 1000 r/min for 5 min. Subsequently, EP tubes were digested for the second time with 0.2% type II collagenase (Solarbio) at 37 °C for 4 h. Undigested tissue pieces were filtered out using a cell sieve (70 μm). The cell density was adjusted to 1 × 10^5^ /ml and incubated at 37 °C in a 5% CO_2_ incubator. Cell passages were performed with a fusion rate of 80% or more. The Y-NPC and O-NPC of P3 were chosen for subsequent experiments because they are relatively stable in synthesizing extracellular matrix. In addition, human normal NPC (N-NPC; Cat NO.: CP-H097) was purchased from Wuhan Pricella Life Technology Co.

Toluidine blue staining was used for the identification of N-NPC, Y-NPC and O-NPC. Briefly, 4% paraformaldehyde (Beyotime, China) was used to fix the cells at room temperature for 15 min. The slides were washed 3 times with distilled water for 2 min each. Toluidine blue staining solution (Solarbio) was added to the slides and incubated at room temperature for 2 min. Subsequently, the slices were sealed using neutral gum and pictures were acquired under a BX53 light microscope (Olympus, Janpan).

### Axial stress treatment

The FX-5000 C™ Compression System (Flexcell International, USA) was applied to the N-NPC and Y-NPC with axial stress. As previously described [25–27], the NPC was digested by trypsin and mixed with 1.2% alginate solution (Aladdin, China). The NPC suspension was added to Flexcell plates (6-well plates) containing 0.1 mol/L CaCl_2_ solution (Aladdin) and gel beads at a concentration of 2 × 10^5^/well. After 15 min of incubation, the gel beads in Flexcell plates were washed with 0.15 mol/L NaCl solution (Aladdin). Flexcell plates were placed in a pressurized culture system and the pressure was adjusted to 1 mPa twice daily for 2 h for 7 days of stimulation. After stress stimulation, the gel beads were dissolved with 1 mL of 3.55 mM sodium citrate solution containing 0.15 mol/L NaCl solution to obtain cells. Control cells were placed in a pressurized culture system with no stress applied.

### Cell proliferation and apoptosis assay

Cell viability of N-NPC, Y-NPC and O-NPC was detected using CCK8 kit (Dojindo, Janpan). Briefly, the concentration of each NPC was adjusted to 5 × 10^5^/ml and inoculated in 96-well plates. A blank control well was set up, and 3 replicate wells were set up for the experimental group. After each NPC was incubated for 0, 12, 24, 48 and 72 h, 10 µl CCK8 solution was added to each well and incubated in the incubator for 4 h. Subsequently, the optical density (OD) of each well was measured at 450 nm using a ELX800 microplate reader (Biotech, USA).

Apoptosis ratios of N-NPC and Y-NPC were detected using ANNEXIN V- FITC/PI Apoptosis Detection Kit (Solarbio). According to the instructions, 5 µl FITC and 10 µl PI were added to N-NPC and Y-NPC after trypsin digestion and incubated for 15 and 5 min, respectively, in the dark. Subsequently, the proportion of cell apoptosis in each group was detected using a Novocyte Advanteon flow cytometer (Agilent, USA).

### Western blotting assay

Total protein was extracted using RIPA lysate (Beyotime), and total protein concentration was determined by EzDrop1000 UV spectrophotometer (BLUE-RAY, China). The total proteins were separated by electrophoresis on SDS-PAGE gel, and transferred to methanol-activated PVDF membrane by wet transfer method. Skim milk powder (5%; Wondersun, China) was applied to seal the PVDF membrane at room temperature for 4 h. Each PVDF membrane was spiked with Rabbit Anti-Wnt3a antibody (bs-1700R; 1:3000; Bioss, China), Rabbit Anti-beta Catenin mAb (ab32572; 1:7000; Abcam, UK), Rabbit Anti-Caspase-3 mAb (ab184787; 1:2000; Abcam), Rabbit Anti-Bcl-2 pAb (ab196495; 1:2000; Abcam), Rabbit Anti-Bax mAb (ab182733; 1:2000; Abcam) and Mouse Anti-beta actin (ab182733; 1:2000; Abcam). Anti-Bcl-2 pAb (ab196495; 1:2000; Abcam), Rabbit Anti-Bax mAb (ab182733; 1:2000; Abcam) and Mouse Anti-β actin mAb (TA-09; 1:4000; ZSGB-BIO, China), and incubated overnight on a SLK-R3000-S shaker (Scilogex, USA) at 4 °C. Then, the PVDF membrane was loaded with Goat Anti Mouse IgG-HRP (M21001L; 1:4000; Abmart, China) or Goat Anti Rabbit IgG-HRP (M21002L; 1:4000; Abmart), and incubated at room temperature for 2 h. PVDF membranes were color developed by ECL Substrate Kit (Abcam), and images were acquired by 5200-multi gel imaging system (Tanon, China). Image J software was used for gray value analysis, and β-actin was used as an internal reference to calculate the relative expression levels of the target proteins.

### RT-qPCR assay

Total RNA from each group of NPCs was extracted using Trizol Reagent (Life Technologies, USA), and 1 µl of total RNA samples was taken to determine the concentration on an EzDrop 1000 UV spectrophotometer (BLUE-RAY). Total RNA was reverse transcribed to cDNA using FastKing RT Kit (With gDNase) (TIANGEN, Germany), and the target gene was amplified using Taq Pro Universal SYBR qPCR Master Mix (Vazyme, China) in 7500 Real-Time PCR System (ABI, USA). The total RNA concentration of each group was shown in Table 2. PCR reaction system (10 µl) consists of Taq Pro Universal SYBR qPCR Master Mix (5 µl), upstream and downstream primers (0.25 µl each), cDNA (1 µl) and nuclease-free water (3.5 µl). Thermal cycle conditions were denaturation at 95 °C for 1 min, annealing at 60 °C for 1 min and extension at 72 °C for 30 s, with a total of 35 cycles. The primer sequences of each gene were GAPDH-F: TTGCCCTCAACGACCACTTT, GAPDH-R: TGGTCCAGGGGTCTTACTCC, Wnt3a-F: CACTCGGATACTTCTTACTC, Wnt3a-R: CCAGGGAGGAATACTGTG, β-catenin-F: CCAGTGGATTCTGTGTTG and β-catenin-R: GGCAGTCTGTCGTAATAG. Since GAPDH was demonstrated to be stably expressed in NPC, we chose it as an internal reference [28]. The relative expression levels of target genes were calculated by the 2^−ΔΔCt^ method [29].

**Table 2:** Information of total RNA

Group	Concentration (µg/µl)	260/280
N-NPC	0.731	1.85
	0.418	1.92
	0.731	1.86
N-NPC + F	0.370	1.88
	0.452	1.83
	0.718	1.81
Y-NPC	0.601	1.87
	0.748	1.93
	0.745	1.84
Y-NPC + F	0.743	1.82
	0.747	1.90
	0.540	1.94

### Statistical analysis

All experiments were repeated at least three times. Data were expressed as mean ± standard deviation, and analyzed by GraphPad Prism software. The Kolmogorov-Smirnov test was conducted to determine whether the data conformed to normality. Student’s t-test or one-way variance was used to analyze data that conformed to a normal distribution with no significant differences in variance between groups, and Mann-Whitney U-test or Kruskal-Walli’s test was used for data that did not conform analysis. *P* < 0.05 was considered statistically significant.

## Result

### The difference between NPCs of different ages

**Fig. 1:**
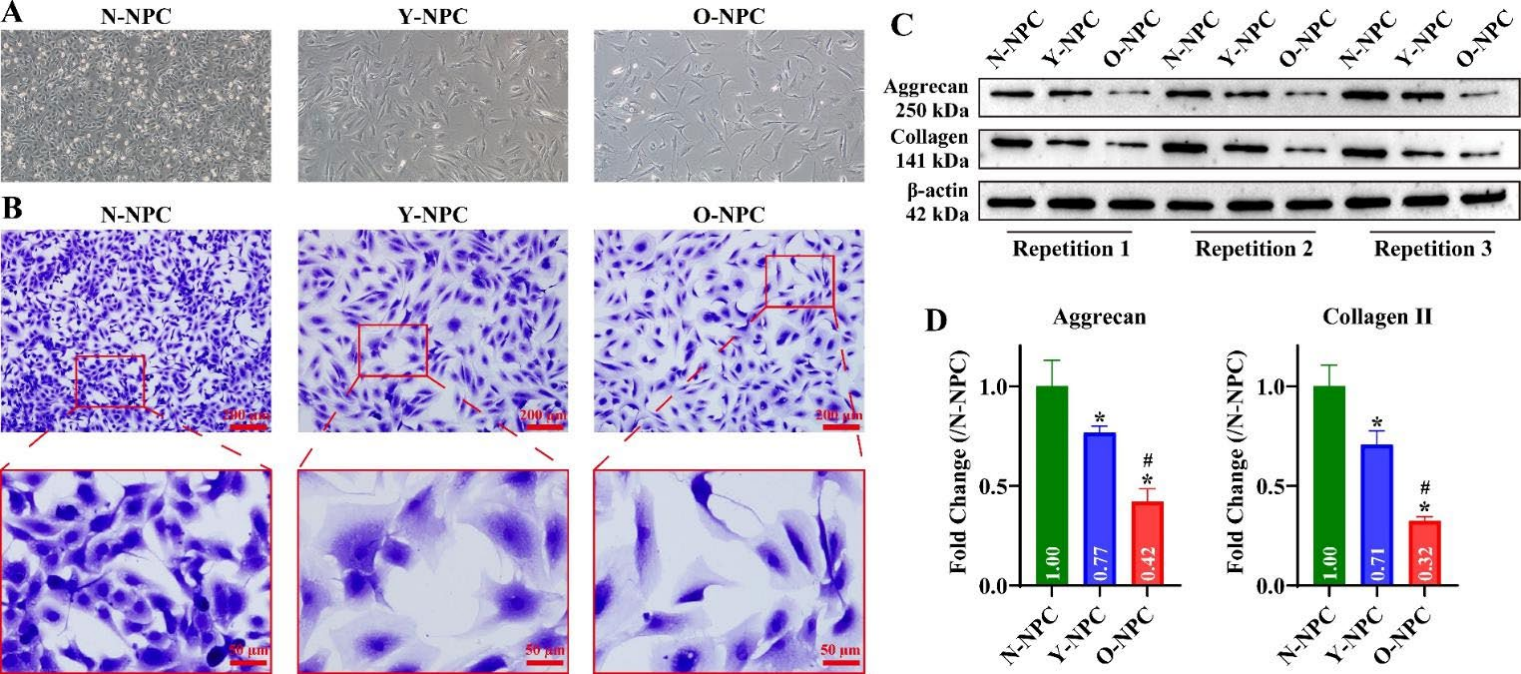
Identification of N-NPC, Y-NPC and O-NPC. A: Representative cell images of N-NPC, Y-NPC and O-NPC (P3). Original magnification: 100 ×. B: The ability of N-NPC, Y-NPC and O-NPC (P3) to synthesize proteoglycans was identified by toluidine blue staining. Scale bar: 200 or 50 μm. C: Representative gel blots of NPC markers Collagen II and Aggrecan. D: Statistical analysis of Collagen II and Aggrecan grayscale values. Compared to the N-NPC group, ^*^P < 0.05; compared to the Y-NPC group, ^#^*P* < 0.05

To understand the activity of the Wnt/β-Catenin pathway in different NPCs, we primary cultured N-NPC, Y-NPC and O-NPC. The N-NPC morphology mainly appears elliptical or polygonal with pseudopods (Fig. 1A). Moreover, the cells were walled, cytoplasm-rich and strongly refractive (Fig. 1A). Y-NPC and O-NPC mainly exhibited shuttle and polygonal shapes with pseudopods and low cell density (Fig. 1A). Notably, O-NPC is less dense and has a flatter morphology than Y-NPC (Fig. 1A). As shown in Fig. 1B, the cytoplasm of N-NPC, Y-NPC and O-NPC were blue, and the coloration was darker near the nucleus. This indicates that the isolated cells are capable of synthesizing proteoglycans. Further, we assayed the expression of NPC markers Collagen II and Aggrecan. The results revealed that all three NPC cells could express Collagen II and Aggrecan (Fig. 1C). Interestingly, Collagen II and Aggrecan had the highest expression in N-NPC, and the lowest expression in O-NPC (Fig. 1D, P < 0.05). Hint, we successfully isolated Y-NPC and O-NPC, and the older the age, the worse the activity and function of NPC.

### Wnt/β-Catenin pathway activity in NPC increases with age

**Fig. 2:**
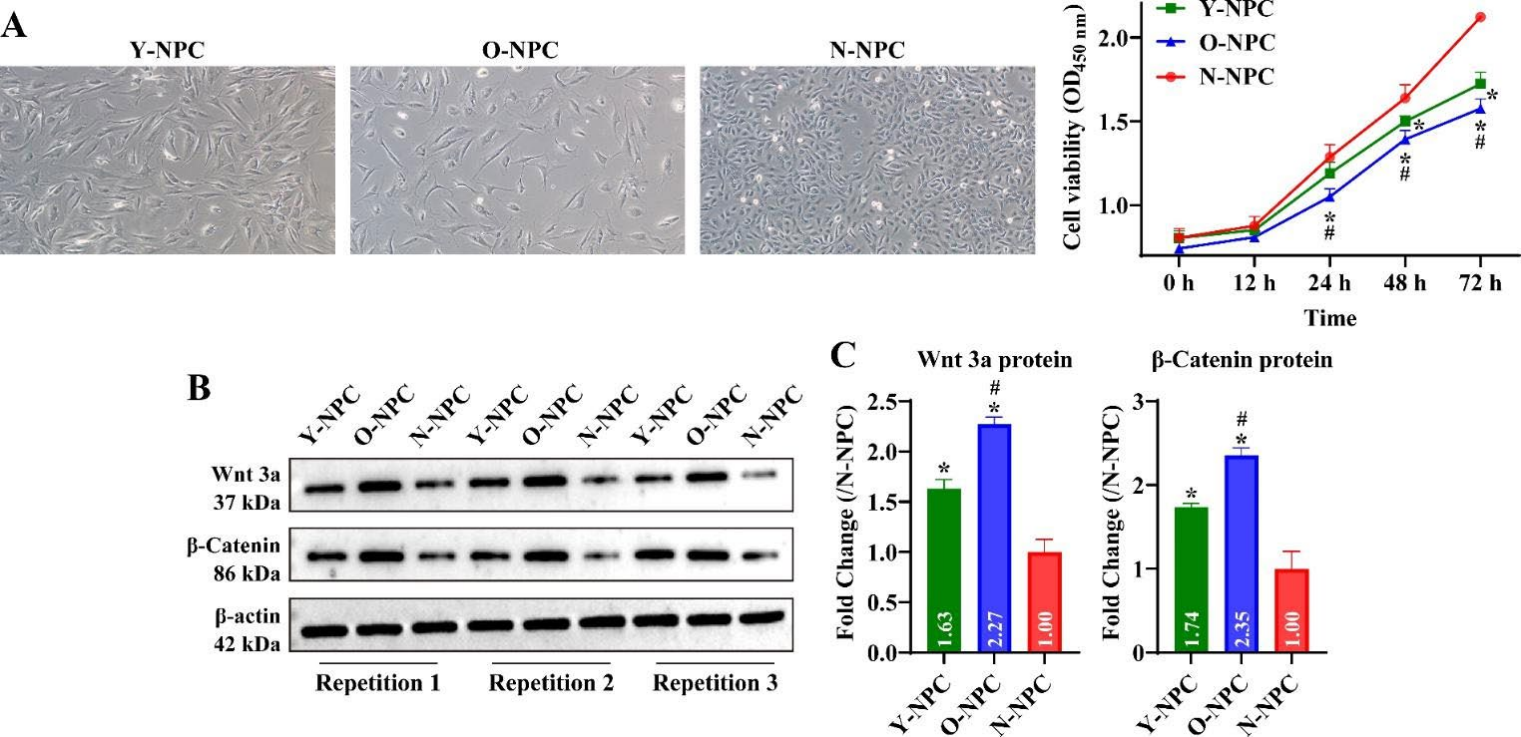
Changes in Wnt/β-Catenin pathway activity in different NPCs. A: The cell viability of N-NPC, Y-NPC and O-NPC (P3) was detected by CCK-8. Representative cell images display the cell states of N-NPC, Y-NPC and O-NPC at 72 h. Original magnification: 100 ×. B: Western blotting was performed to detect the expression of Wnt 3a and β-Catenin in N-NPC, Y-NPC and O-NPC (P3). C: Statistical analysis of changes in Wnt 3a and β-Catenin expression in each group. Compared to the N-NPC group, ^*^P < 0.05; compared to the Y-NPC group, ^#^*P* < 0.05

Further, we explored the changes in the activity of the Wnt/β-Catenin pathway in different NPCs. First, we assayed the cellular activity of three NPCs. The results displayed that at 24 h, the cell viability of O-NPC was significantly smaller than that of N-NPC and Y-NPC; from 48 h, the cell viability of N-NPC was significantly larger than that of both Y-NPC and O-NPC, and the cell viability of Y-NPC was larger than that of O-NPC (Fig. 2A, P < 0.05). In addition, the expression of Wnt 3a and β-Catenin was significantly increased in Y-NPC and O-NPC compared to N-NPC, and the expression of Wnt 3a and β-Catenin was significantly lower in Y-NPC than in O-NPC (Fig. 2B C, P < 0.05). It was suggested that the proliferation of NPC gradually decreased with increasing age, while the Wnt/β-Catenin pathway activity gradually increased.

### Overloaded axial stress facilitates apoptosis and Wnt/β-Catenin pathway activation in NPC

**Fig. 3:**
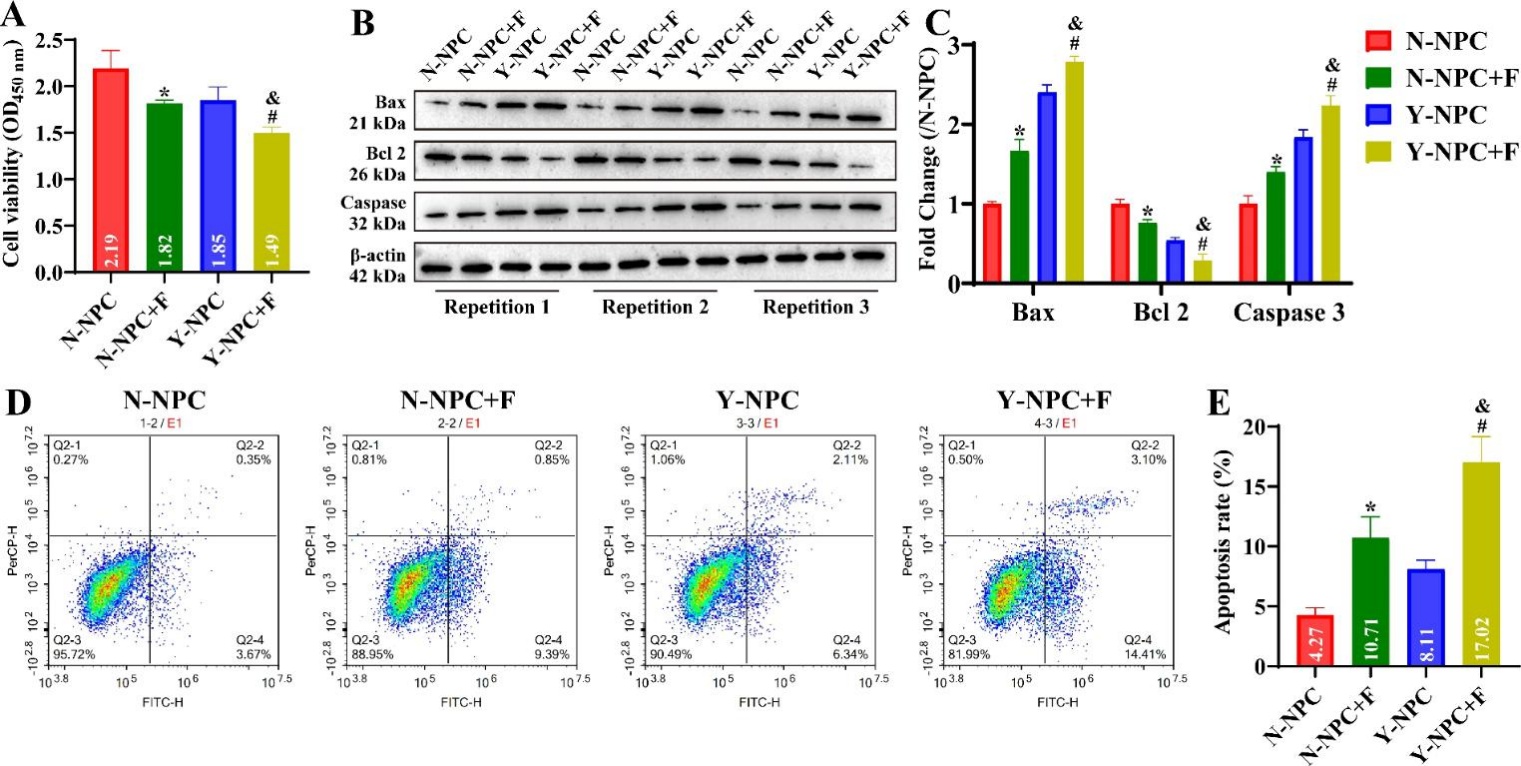
Effect of axial stress on proliferation and apoptosis of N-NPC and Y-NPC. A: Cell viability of N-NPC and Y-NPC (P3) at 72 h before and after axial stress treatment. B-C: The expression of apoptosis-related proteins Bax, Bcl 2 and Caspase in N-NPC and Y-NPC (P3) was detected by Western blotting. D: Effects of axial stress on apoptosis of N-NPC and Y-NPC (P3) detected by flow cytometry. Compared to the N-NPC group, ^*^P < 0.05; compared to the Y-NPC group, ^#^*P* < 0.05; compared to the N-NPC + F group, ^&^*P* < 0.05

**Fig. 4:**
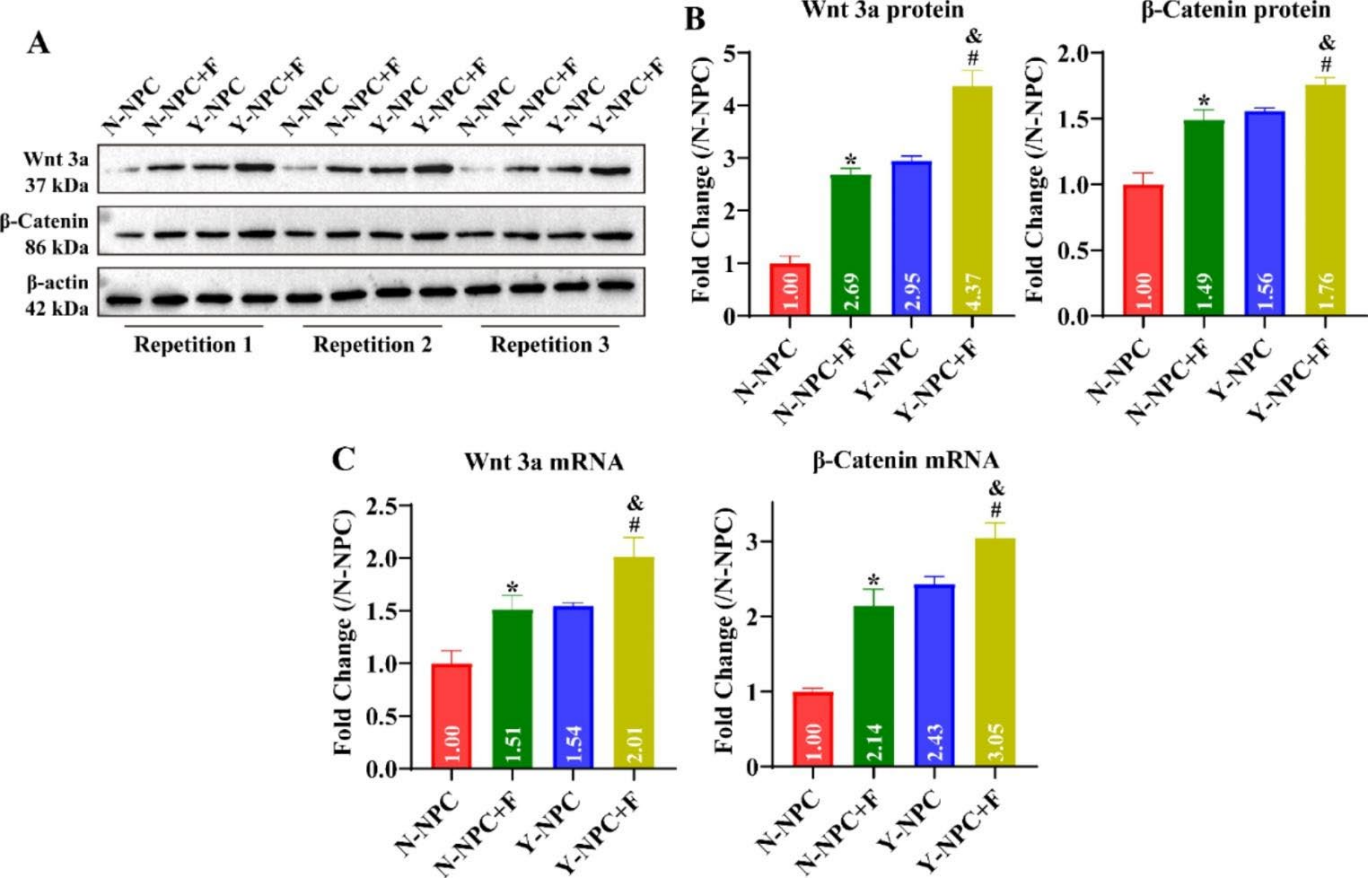
Effect of axial stress on Wnt/β-Catenin pathway activity in N-NPC and Y-NPC. A: Representative gel blots of Wnt 3a and β-Catenin proteins in N-NPC and Y-NPC (P3). B: Statistical analysis of Wnt 3a and β-Catenin protein expression in each group. C: Effect of axial stress on Wnt 3a and β-Catenin mRNA expression in N-NPC and Y-NPC (P3) examined by RT-qPCR. Compared to the N-NPC group, ^*^P < 0.05; compared to the Y-NPC group, ^#^*P* < 0.05; compared to the N-NPC + F group, ^&^*P* < 0.05

We further investigated the effects of axial stress on the proliferation, apoptosis and Wnt/β-Catenin pathway activity of NPC. Since the state of O-NPC is very poor after applying axial stress, this may lead to large errors in the results. Therefore, we excluded exploring the effect of overloaded axial stress on O-NPC. CCK-8 results showed that the cell viability of N-NPC and Y-NPC was significantly reduced after axial stress treatment, and the cell viability of Y-NPC was significantly lower than that of N-NPC (Fig. 3A, P < 0.05). Moreover, we detected changes in the expression of apoptosis-associated proteins. The results indicated that axial stress significantly enhanced the expression of Bax and Caspase 3, and decreased the expression of Bcl 2 in N-NPC and Y-NPC (Fig. 3B C, P < 0.05). Compared to the N-NPC + F group, the expression of Bax and Caspase 3 was significantly increased, and the expression of Bcl 2 was significantly decreased in the Y-NPC + F group (Fig. 3B C, P < 0.05). Consistently, the apoptosis ratio of N-NPC and Y-NPC significantly expanded from 4.27 ± 0.5 and 8.11 ± 0.62 to 10.71 ± 1.44 and 17.02 ± 1.73 after axial pressure treatment, respectively (Fig. 3D and E, P < 0.05). Western blotting results showed that the expression levels of Wnt 3a and β-Catenin proteins were significantly increased in N-NPC and Y-NPC after axial stress treatment, and were significantly higher in the Y-NPC + F group than in the N-NPC + F group (Fig. 4A and B, P < 0.05). RT-qPCR results revealed that the trends of Wnt 3a and β-Catenin mRNA were consistent with the protein (Fig. 4C and D, P < 0.05). This demonstrates that axial stress inhibits NPC proliferation, and promotes its apoptosis and Wnt/β-Catenin pathway activation.

## Discussion

In ADS patients, a series of degenerative changes involving the centrum, intervertebral discs, facet joints, and paravertebral ligaments can be observed. The degenerative changes are progressively aggravated, leading to deformities in appearance and a variety of symptoms such as low back pain, radiating pain in the lower extremities and intermittent claudication, which seriously affect the daily life of patients. Biomechanical factors were found to play an important role in IVDD caused by scoliosis and disc wedging [30, 31]. In the present study, we investigated the effect of axial pressure on the Wnt/β-Catenin pathway in NPC of ADS-IVDD. We found that older patients had lower proteoglycan and collagen II expression, and stronger Wnt/β-Catenin pathway activity in NPC of ADS-IVDD patient-derived NPCs. Furthermore, axial pressure inhibits the proliferation of different NPCs and contributes to their apoptosis, which is achieved by activating the Wnt/β-Catenin pathway.

The Wnt/β-Catenin pathway is significant in stem cell renewal, cell proliferation and cell differentiation during both embryogenesis and adult tissue homeostasis. In the classical Wnt/β-catenin pathway, active β-catenin accumulates in the cytoplasm and translocates to the nucleus, which activates factors such as cytosolic transcription factors and lymphatic enhancers to initiate transcription of downstream target genes. In recent years, there is increasing evidence that this signaling pathway also plays an important role in the anabolism of cartilage [17]. Miyamoto et al. [32] reported that cartilage degeneration could be delayed by inhibiting the Wnt/β-Catenin pathway. Ursula et al. [33] demonstrated that activation of the Wnt/β-Catenin pathway can inhibit chondrocyte differentiation. As a chondrocyte-like cell, NPCs are highly consistent with chondrocytes in terms of gene expression and function. In fact, it has been shown that the Wnt/β-Catenin pathway is aberrantly activated in IVDD and inhibits NPC proliferation and suppresses its apoptosis [19, 34]. We found that the results were equally applicable in ADS-IVDD patient-derived NPC. Clinical studies have confirmed that IVD degenerates to varying degrees with age [35]. Basic research has confirmed that as the body ages, the number of cells and proteoglycan content in IVD tissues continue to decrease, leading to a reduction in hydration and subsequently inducing a diminution in elasticity and load resistance [36]. Similarly, in our study, the ability of O-NPC to synthesize Collagen II and Aggrecan was decreased compared to N-NPC and Y-NPC, which is consistent with previous studies. In addition, we found that the Wnt/β-Catenin pathway activity was higher in older NPCs. Mechanistically, this may be due to the activation of the Wnt/β-Catenin pathway facilitating the gene expression of MMPs and ADAMTs, which results in the degradation of Collagen II and Aggrecan in the ECM [37]. Abnormal mechanical stimulation is one of the important factors contributing to the pathogenesis of IVDD, but the specific mechanism by which it induces the onset and development of IVDD remains unclear. In the present study, we found that overloaded axial stress was able to further activate Wnt/β-Catenin pathway in NPC. In addition, the overloaded axial stress could further inhibit NPC proliferation and contribute to its apoptosis. Taken together with previous studies, we believe that the effect of overloaded axial stress on NPC proliferation and apoptosis is achieved through activation of the Wnt/β-Catenin pathway.

Although our findings are positive, there are still many limitations. For example, we lack in vivo experiments to verify the effect of overloaded axial stress on Wnt/β-Catenin pathway activity in ADS-IVDD. In addition, the upstream and downstream targets of the overloaded axial stress-regulated Wnt/β-Catenin pathway remain to be investigated. In conclusion, our study confirmed that overloaded axial pressure inhibits the proliferation and facilitates the apoptosis of ADS-IVDD patient-derived NPCs by activating the Wnt/β-Catenin pathway, which provides a theoretical basis for future prevention and treatment of ADS-IVDD.

## Electronic supplementary material

Below is the link to the electronic supplementary material.

Supplementary Material 1

## Data Availability

All the data obtained and materials analyzed in this research are available with the corresponding author upon reasonable request.

## References

[1] York PJ, Kim HJ (2017). Degenerative Scoliosis[J]. Curr Rev Musculoskelet Med.

[2] Diebo BG, Shah NV, Boachie-Adjei O (2019). Adult spinal deformity[J]. Lancet.

[3] Mcaviney J, Roberts C, Sullivan B (2020). The prevalence of adult de novo scoliosis: a systematic review and meta-analysis[J]. Eur Spine J.

[4] Wise CA, Sepich D, Ushiki A (2020). The cartilage matrisome in adolescent idiopathic scoliosis[J]. Bone Res.

[5] Kim W, Porrino JA, Hood KA (2019). Clinical evaluation, imaging, and management of adolescent idiopathic and adult degenerative Scoliosis[J]. Curr Probl Diagn Radiol.

[6] De Reuver S, Moens A, Kruyt MC (2022). Ultrasound Shear Wave Elastography of the intervertebral disc and idiopathic scoliosis: a systematic Review[J]. Ultrasound Med Biol.

[7] Cristante AF, Silva RTE, Costa G (2021). Adult degenerative Scoliosis[J]. Rev Bras Ortop (Sao Paulo).

[8] Lippross S, Girmond P, Lüders KA et al (2021) Smaller intervertebral disc volume and more disc degeneration after spinal distraction in Scoliotic Children[J].J Clin Med, 10(10) 10.3390/jcm10102124 PMC8156152 34068964

[9] Oichi T, Taniguchi Y, Oshima Y (2020). Pathomechanism of intervertebral disc degeneration[J]. JOR Spine.

[10] Kos N, Gradisnik L, Velnar T (2019). A brief review of the degenerative intervertebral disc Disease[J]. Med Arch.

[11] Desmoulin GT, Pradhan V, Milner TE (2020). Mechanical aspects of intervertebral disc Injury and Implications on Biomechanics[J]. Spine (Phila Pa 1976).

[12] Tendulkar G, Chen T, Ehnert S et al (2019) Intervertebral disc Nucleus Repair: hype or hope?[J].Int J Mol Sci, 20(15) 10.3390/ijms20153622 PMC6696292 31344903

[13] Yamada K, Iwasaki N, Sudo H (2022) Biomaterials and cell-based regenerative therapies for intervertebral disc degeneration with a focus on Biological and Biomechanical Functional Repair: targeting treatments for disc Herniation[J].Cells, 11(4) 10.3390/cells11040602 PMC8870062 35203253

[14] Li B, Yang Y, Wang L et al (2021) Stem Cell Therapy and Exercise for Treatment of Intervertebral Disc Degeneration[J]. Stem Cells Int, 2021: 7982333 10.1155/2021/7982333 PMC8528633 34691192

[15] Liang H, Luo R, Li G et al (2022) The proteolysis of ECM in intervertebral disc Degeneration[J].Int J Mol Sci, 23(3) 10.3390/ijms23031715 PMC8835917 35163637

[16] Liu J, Xiao Q, Xiao J (2022). Wnt/β-catenin signalling: function, biological mechanisms, and therapeutic opportunities[J]. Signal Transduct Target Ther.

[17] Huang P, Yan R, Zhang X (2019). Activating Wnt/β-catenin signaling pathway for disease therapy: Challenges and opportunities[J]. Pharmacol Ther.

[18] Hiyama A, Sakai D, Risbud MV (2010). Enhancement of intervertebral disc cell senescence by WNT/β-catenin signaling-induced matrix metalloproteinase expression[J]. Arthritis Rheum.

[19] Hao Y, Ren Z, Yu L (2022). p300 arrests intervertebral disc degeneration by regulating the FOXO3/Sirt1/Wnt/β-catenin axis[J]. Aging Cell.

[20] Wang JJ, Liu XY, Du W (2020). RBMS3 delays disc degeneration by inhibiting Wnt/β-catenin signaling pathway[J]. Eur Rev Med Pharmacol Sci.

[21] Liu W, Wang Y (2021). Protective role of the alpha-1-antitrypsin in intervertebral disc degeneration[J]. J Orthop Surg Res.

[22] Ge J, Cheng X, Yan Q (2020). Calcitonin inhibits intervertebral disc degeneration by regulating protein kinase C[J]. J Cell Mol Med.

[23] Niu Z, Tang G, Wang X (2023). Trigonochinene E promotes lysosomal biogenesis and enhances autophagy via TFEB/TFE3 in human degenerative NP cells against oxidative stress[J]. Phytomedicine.

[24] Arai F, Hiyama A, Sakai D (2012). The expression and role of non-canonical (PKC) signaling in nucleus pulposus cell metabolism[J]. J Orthop Res.

[25] Swiatlowska P, Sit B, Feng Z (2022). Pressure and stiffness sensing together regulate vascular smooth muscle cell phenotype switching[J]. Sci Adv.

[26] Li J, Ma Y, Jiao Y et al (2022) Intervertebral Disc Degeneration and Low Back Pain Depends on Duration and Magnitude of Axial Compression[J]. Oxid Med Cell Longev, 2022: 1045999 10.1155/2022/1045999 PMC9076309 35528509

[27] Song M, Zhang Y, Sun Y (2022). Inhibition of RhoA/MRTF-A signaling alleviates nucleus pulposus fibrosis induced by mechanical stress overload[J]. Connect Tissue Res.

[28] Yurube T, Takada T, Hirata H (2011). Modified house-keeping gene expression in a rat tail compression loading-induced disc degeneration model[J]. J Orthop Res.

[29] Livak KJ, Schmittgen TD (2001). Analysis of relative gene expression data using real-time quantitative PCR and the 2(-Delta Delta C(T)) Method[J]. Methods.

[30] Peng Y, Wang SR, Qiu GX (2020). Research progress on the etiology and pathogenesis of adolescent idiopathic scoliosis[J]. Chin Med J (Engl).

[31] O’leary SA, Paschos NK, Link JM (2018). Facet joints of the spine: structure-function Relationships, problems and treatments, and the potential for Regeneration[J]. Annu Rev Biomed Eng.

[32] Miyamoto K, Ohkawara B, Ito M (2017). Fluoxetine ameliorates cartilage degradation in osteoarthritis by inhibiting Wnt/β-catenin signaling[J]. PLoS ONE.

[33] Kreuser U, Buchert J, Haase A (2020). Initial WNT/β-Catenin activation enhanced mesoderm commitment, Extracellular Matrix expression, cell aggregation and cartilage tissue yield from Induced Pluripotent Stem Cells[J]. Front Cell Dev Biol.

[34] Yang X, Sun Y, Li X (2022). Rac1 regulates nucleus pulposus cell degeneration by activating the Wnt/β-catenin signaling pathway and promotes the progression of intervertebral disc degeneration[J]. Am J Physiol Cell Physiol.

[35] Li Y, Gao L, Meng C (2022). The order of degeneration of lumbar intervertebral disc and lumbar facet joint and its correlation with age[J]. Minerva Med.

[36] Wang F, Cai F, Shi R (2016). Aging and age related stresses: a senescence mechanism of intervertebral disc degeneration[J]. Osteoarthritis Cartilage.

[37] Hiyama A, Sakai D, Tanaka M (2011). The relationship between the Wnt/β-catenin and TGF-β/BMP signals in the intervertebral disc cell[J]. J Cell Physiol.

