# Supplementary figures and images for: Overloaded axial stress activates the Wnt/β-Catenin pathway in nucleus pulposus cells of adult degenerative scoliosis combined with intervertebral disc degeneration
Source: Mol Biol Rep. 2023 Apr 8;50(6):4791–8. doi: 10.1007/s11033-023-08390-9 (PMC10209306; doi:10.1007/s11033-023-08390-9)

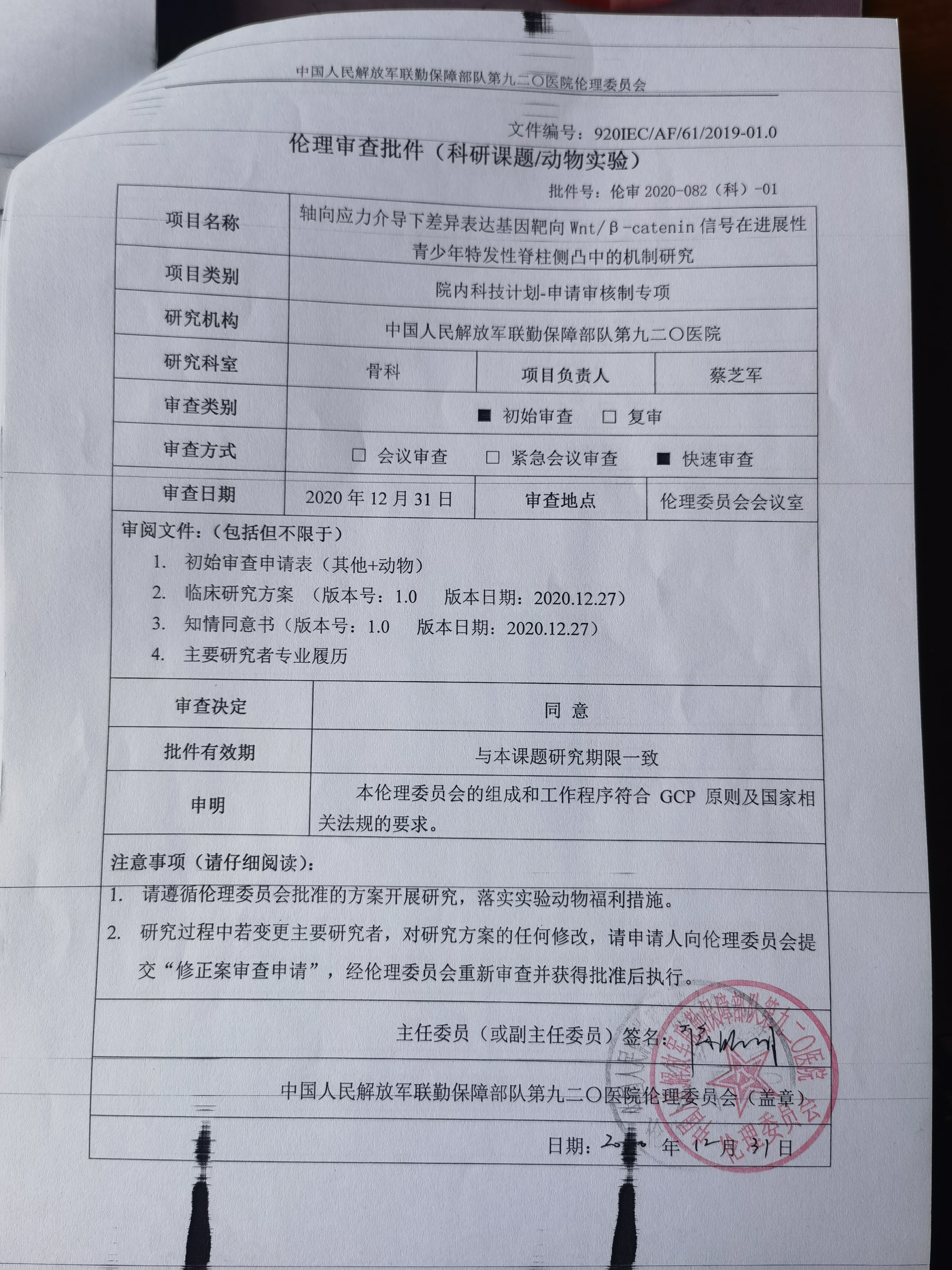

Supplement: Supplementary file 1 — Supplementary Material 1 [file 11033_2023_8390_MOESM1_ESM.jpg]
